# Protective Role of Ramipril and Candesartan against Myocardial Ischemic Reperfusion Injury: A Biochemical and Transmission Electron Microscopical Study

**DOI:** 10.1155/2016/4608979

**Published:** 2016-03-06

**Authors:** Mohamed Saleem Thattakudian Sheik Uduman, Rajitha Bodd Reddy, Priyanka Punuru, Gopinath Chakka, Gauthaman Karunakaran

**Affiliations:** ^1^Department of Pharmacology, Annamacharya College of Pharmacy, Rajampet 516126, India; ^2^Department of Pharmacology, College of Pharmacy, King Khalid University, Abha 61441, Saudi Arabia

## Abstract

The present study was designed to investigate the role of combined administration of Ramipril and Candesartan against* in vitro *myocardial ischemic reperfusion injury in rat. Male Wistar albino rats were divided into five groups (*n* = 6) and treated with saline (10 mL/kg), Ramipril (2 mg/kg), Candesartan (1 mg/kg), and the combination of both drugs, respectively 24 h before induction of global ischemia (5 min of stabilization, 9 min of global ischemia, and 12 min of reflow). Combination of Ramipril and Candesartan when compared to the monotherapy significantly increased the levels of superoxide dismutase, reduced glutathione, catalase, and nitric oxide and decreased the levels of thiobarbituric acid reactive substances. In addition, the superior protective role of combination of Ramipril and Candesartan on ischemia induced myocardial damage was further confirmed by well preserved myocardial tissue architecture in light microscopy and transmission electron microscopy analysis studies. The combination was proved to be effective in salvaging the myocardial tissue against ischemic reperfusion injury when compared to the monotherapy of individual drugs and further investigations on protective mechanism of drugs by increasing the nitric oxide level at molecular levels are needed.

## 1. Introduction

In spite of the advances in the cardiovascular disease (CVD), ischemic heart disease (IHD) is one of the leading causes of death in the world. According to the World Health Organization (WHO), 7,254,000 deaths worldwide (12.8% of all deaths) resulted from IHD in 2008 [1]. Acute myocardial ischemia reperfusion injury (MIRI) is the major cause of the detrimental effects of IHD on the myocardium [2]. MIRI occurs during the invasive treatments such as, thrombolysis, angioplasty, coronary bypass, and heart transplantation [3]. The treatment for acute myocardial infarction is the use of thrombolytic therapy or primary percutaneous coronary intervention (PCI). But these treatments cause myocardial reperfusion injury for which there is no effective therapy [4].

Angiotensin converting enzyme (ACE) converts angiotensin I (Ang I) to angiotensin II (Ang II). An increase in Ang II is deleterious in the setting of MIRI. At pathophysiological levels, Ang II induces myocardial necrosis, promotes cardiac hypertrophy, positive inotropism, and increases cardiac levels of norepinephrine, resulting in increased arrhythmogenicity and coronary vasoconstriction [5].

ACE inhibitors have demonstrated significant clinical benefit by decreasing the levels of circulating Ang II by inhibiting ACE [6]. But, in experimental models, they have not been as effective as expected in attenuating reperfusion injury, because of the presence of ACE independent enzymes, such as heart chymase that converts Ang I to Ang II. Angiotensin II receptor blockers (ARBs) act by selectively blocking angiotensin I (AT_1_) receptor, thereby directly blocking the vasoconstrictor and growth effects of Ang II [7].

Activation of AT_2_ receptor mediates the release of bradykinin and the activation of nitric oxide release [8]. AT_1_ receptor inhibition with ARBs alone is not sufficient to suppress renin angiotensin system activity because it leaves AT_2_ receptor open for stimulation by alternatively formed Ang II. The same is true for ACE inhibition due to counter-regulatory pathways related to plasma renin activity (PRA). As a result, the combination of ARBs and ACE inhibitors might produce a more complete inhibition of the system and enhance bradykinin accumulation resulting in increased endothelial nitric oxide (NO) production [9].

More research evidence was available in the use of ARBs in the prevention of CVD. Independent activation of AT_1_ receptor involved in the development of pathological changes in the cardiac muscles [10]. Few earlier studies demonstrated that Candesartan [11], Ramipril [12] individually showed cardioprotective effects against MIRI. But no reports were available on Ramipril in combination with Candesartan on* in vitro* model of MIRI. Hence, the aim of the present study was designed to evaluate the role of Ramipril in combination with Candesartan on* in vitro* model of MIRI.

## 2. Materials and Methods

### 2.1. Animals

30 male Wistar albino rats, weighing between 200 and 250 g, were included in the study. Rats were housed in the departmental animal house at an ambient temperature of 25°C, under a 12-hour dark-12-hour light cycle for the whole period of the study. The rats were randomly assigned to five groups with *n* = 6 each as follows: (1) control, (2) ischemic control (I/R), (3) Ramipril (2 mg/kg), (4) Candesartan (1 mg/kg), and (5) Ramipril (2 mg/kg) + Candesartan (1 mg/kg). All groups were fed with standard pellet diet with tap water* ad libitum*. Experiments were carried out according to the guidelines given by the committee for the purpose of control and supervision of experiments on animals (CPCSEA), New Delhi (India) and the protocol was approved by the Institutional Animal Ethics Committee (1220/a/08/CPCSEA).

### 2.2. Drug Administration

The respective drugs were given 24 hours before sacrificing the animals. After 24 hours, the rats were heparinised (375 units/200 g i.p) [13], and half an hour later, rats were anaesthetised with ether and subjected to the protocol below.

### 2.3. *In Vitro* MIRI

Rats from each group except the control group were anaesthetised with ether, skin was incised, and cut was made on the chest to expose the heart. Then, heart along with one cm of ascending aorta attached was quickly removed and dipped in ice-cold saline. The hearts were then mounted on Langendorff apparatus and perfused with Henseleit (K-H) buffer at a constant pressure of 60–70 mmHg at 37°C and aerated with a mixture of O_2_ (95%) and CO_2_ (5%).

Following an initial period of 5 min of stabilization, the flow is stopped for 9 minutes (ischemia) followed by reperfusion with K-H buffer for 12 minutes (reperfusion) [14–17].

Hearts were detached from Langendorff apparatus and stored in 10% buffered formalin and 2.5% glutaraldehyde solution for histopathology studies and for TEM analysis, respectively. Parts of hearts were stored under freezing conditions for estimations of biochemical parameters.

### 2.4. Estimation of Biochemical Parameters

Hearts tissues were homogenized with 10% trichloroacetic acid (TCA) in 1 : 10 ratio (for 1 gm of tissue 10 mL of 10% TCA was added) and centrifuged at 3000 ×g for 10 min and the supernatant was used for the estimation of thiobarbituric acid reactive substances (TBARS) [18], reduced glutathione (GSH) [19], superoxide dismutase (SOD) [20], catalase (CAT) [21], protein [22], and nitric oxide (NO) [23].

### 2.5. Histopathology Studies

#### 2.5.1. Light Microscopy

The hearts stored in 10% buffered formalin were embedded in paraffin; sections were cut at 5 *μ*m and stained with hematoxylin and eosin. These sections were then examined under a light microscope for histological changes.

#### 2.5.2. Transmission Electron Microscopical (TEM) Analysis

Samples were fixed in 2.5% glutaraldehyde in 0.1 M phosphate buffer (pH 7.2) for 24 hrs at 4°C and washed with PBS for 4 times at an interval of 45 mins and then postfixed in 1% aqueous osmium tetroxide for 2 hours. Later, they were washed with deionised distilled water for 4–6 times at an interval of 45 mins, dehydrated in series of graded alcohols, infiltrated and embedded in araldite 6005 resin, and incubated at 80°C for 72 h for complete polymerization. Ultrathin (50–70 nm) sections were made with a glass knife on ultramicrotome (Leica Ultracut UCT-GA-D/E-1/100), mounted on copper grids, and stained with saturated aqueous uranyl acetate (UA) and counter-stained with Reynolds lead citrate (LC), viewed under TEM (model: Hitachi, H-7500 from Japan), at required magnifications as per the standard procedures.

### 2.6. Statistical Analysis

All values are expressed as mean ± SEM. Statistical analysis carried out by using One-Way ANOVA with Dunnett's posttest was performed using GraphPad Prism version 5.00 for Windows, GraphPad Software, San Diego, CA, USA, http://www.graphpad.com. Significance is set at *p* < 0.05.

## 3. Results

### 3.1. Effect on Oxidative Stress Parameters

TBARS, GSH, SOD, and CAT levels were estimated in myocardial tissue homogenate. The results were represented in Table 1.

### 3.2. Thiobarbituric Acid Reactive Substances

Myocardial TBARS in ischemic control group (117.1 ± 36.8 nmol/g wet wt) was significantly (*p* < 0.001) higher than that in control group (57.3 ± 10.3 nmol/g wet wt). In animals treated with Ramipril, Candesartan, and combination of Ramipril and Candesartan, there was significantly (*p* < 0.001) lower myocardial TBARS levels (58.6 ± 1.1 nmol/g wet wt, 52.2 ± 2.8 nmol/g wet wt, and 43.6 ± 2.7 nmol/g wet wt, resp.) in comparison to ischemic control group.

### 3.3. Reduced Glutathione

Myocardial GSH levels were significantly low (*p* < 0.001) in ischemic control group (55.7 ± 5.9 *μ*g/gm wet wt) in comparison to control group (222.1 ± 5.3 *μ*g/gm wet wt). There was a significant increase (*p* < 0.01) in the levels of GSH in the Ramipril group (61.04 ± 3.3 *μ*g/gm wet wt) and Candesartan group (80.4 ± 10.2 *μ*g/gm wet wt). However, the myocardial GSH levels were significantly (*p* < 0.001) higher (200.2 ± 8.1 *μ*g/gm wet wt) in combination group in comparison to ischemic control group.

### 3.4. Superoxide Dismutase

There was a significant (*p* < 0.05) decrease in myocardial SOD activity in ischemic control group (6.46 ± 0.13 IU/dL) in comparison to that of control group (18.7 ± 6.3 IU/dL). Myocardial SOD levels showed no significant change in the Ramipril and Candesartan groups (9.7  ±  4.9 IU/dL and 13.4  ±  9.5 IU/dL, resp.) in comparison to ischemic control group. However, the myocardial SOD levels were significantly (*p* < 0.001) higher (26.4 ± 8.6 IU/dL) in combination group in comparison to ischemic control group.

### 3.5. Catalase

Myocardial catalase levels were significantly (*p* < 0.001) lower in ischemic control group (5.4 ± 0.55 IU/dL) in comparison to that of control group (21.73 ± 0.59 IU/dL). Myocardial catalase levels showed no significant change in the Ramipril and Candesartan groups (6.04 ± 0.24 IU/dL and 7.7 ± 1.03 IU/dL, resp.) in comparison to IR group, whereas the combination group showed significant (*p* < 0.001) increase (19.8 ± 0.78 IU/dL) in myocardial catalase levels in comparison to ischemic control group.

### 3.6. Effect on Tissue Nitrate Levels

Nitrate levels were estimated in the myocardial tissue homogenate and the results were represented in Table 2. Myocardial tissue nitrate levels were significantly (*p* < 0.01) low in ischemic control group (44 ± 2.3 *μ*g/dL) in comparison to that of control group (52 ± 15.3 *μ*g/dL). There was no significant change in myocardial tissue nitrate levels in Ramipril (48 ± 3.7 *μ*g/dL) and in Candesartan group myocardial tissue nitrate levels were significantly (*p* < 0.05) higher (50 ± 3.6 *μ*g/dL) and also in combination group the NO levels were significantly (*p* < 0.001) higher (55 ± 7.4 *μ*g/dL) compared to ischemic control group.

### 3.7. Results of Light Microscopy Analysis

**Figure 1:**
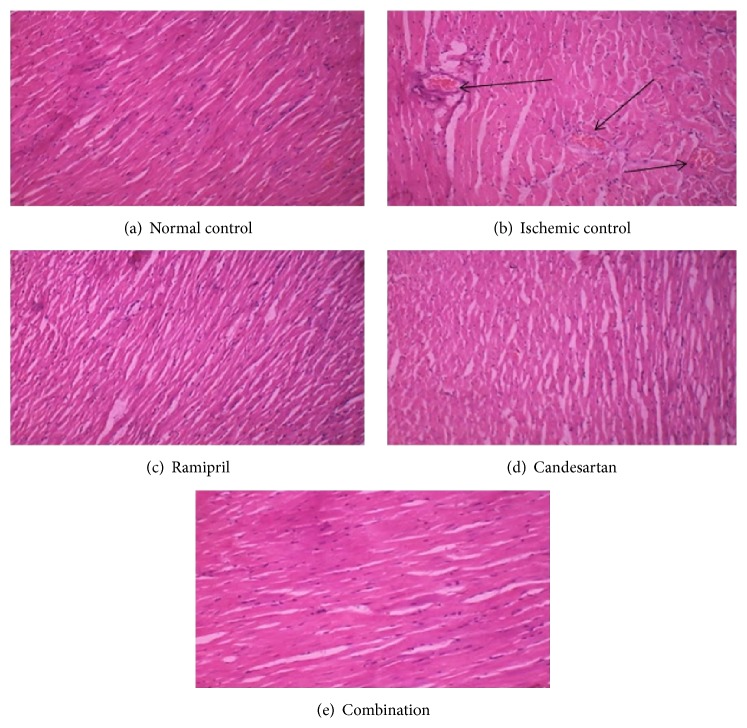
Histopathology of heart. (a) Control rat heart, (b) Control rat heart subjected to IRI, (c) Ramipril treated rat heart subjected to IRI, (d) Candesartan treated rat heart subjected to IRI, and (e) Ramipril and Candesartan treated rat heart subjected to IRI.

Figure 1 shows the extent of histopathological changes in myocardial tissues in vehicle and drug treated rats. In the present study, the tissue sections of control group showed normal myofibrillar structure with striations, branched appearance, and continuity with adjacent myofibrils. Ischemic control group showed extensive degeneration of myofibrils, edema, focal haemorrhage, and leukocyte infiltration which are indicative of necrosis. The tissue sections of Ramipril, Candesartan, and combination groups showed normal myofibrillar structure with striations, branched appearance, and continuity with adjacent myofibrils. In all these three groups, the morphology of cardiac muscle fibers was relatively well preserved.

### 3.8. Results of Transmission Electron Microscopical Study

**Figure 2:**
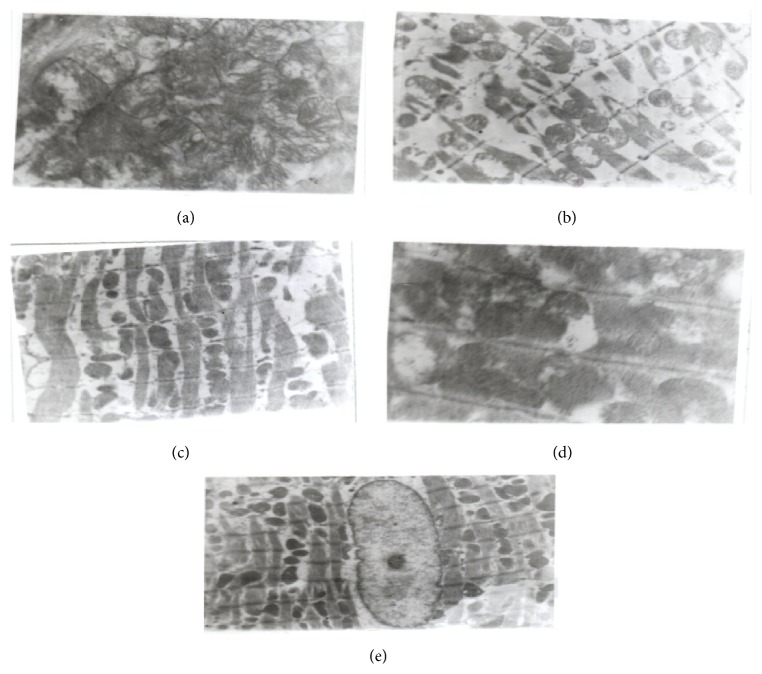
Transmission electron micrograph of rat heart. (a) Control rat heart, (b) Control rat heart subjected to IRI, (c) Ramipril treated rat heart subjected to IRI, (d) Candesartan treated rat heart subjected to IRI, and (e) Ramipril and Candesartan treated rat heart subjected to IRI.

Figure 2 shows the extent of ultrastructural changes in vehicle and drug treated groups. Characteristic changes were seen in the rat heart subjected to IRI (group C-IR). There was significant disruption of myofilament and Z-band architecture in C-IR group. Other ultrastructural changes were manifested by loss of cell membrane integrity, interstitial edema, the appearance of vacuoles within the cell, and changes in the mitochondrial architecture. Extensive loss of crystae and double membrane and presence of vacuoles in mitochondria were prominent. However, myocardial ultrastructure was found to be well preserved and less evidence of myocyte injury was observed in Ramipril, Candesartan, and combination of both drugs treated groups. Only occasional disruption of myofilament, mild interstitial edema, and less accumulation of electron dense material in mitochondria were noticed in Ramipril, Candesartan, and combination of both drugs treated groups.

## 4. Discussion

In the present study, the role of Ramipril in combination with Candesartan was evaluated in rat model of MIRI.

Both ACE inhibitors and ARBs interfere with the activity of the RAAS in a different way. The combination of ACE inhibitors with ARBs could lead to a more effective inhibition of RAAS. Combined RAAS blockade may also prevent the ACE escape phenomenon that decreases the effectiveness of ACE inhibitors as ARBs block all Ang II action at the AT_1_ receptor sites [24]. Ang II activates the enzyme NADPH oxidase (nicotinamide adenine dinucleotide phosphate-oxidase) which oxidases NADPH to NADPH^+^, reducing O_2_ to O_2_^*∗*−^ in the oxidizing process. This superoxide is further involved in the formation of H_2_O_2_ (hydrogen peroxide), ^*∗*^OH (hydroxyl radical), and ONOO^−^ (peroxynitrite) and leads to myocardial tissue damage due to the developed oxidative stress [25]. By preventing the formation of Ang II, the formation of free radicals can be reduced and NO levels can be increased which could be beneficial in the protection of myocardial tissue against MIRI.

Several researches proved the effect of angiotensin inhibition in the ischemic myocardium. The hemodynamic effects of losartan and ramiprilat were well established in the MIRI model of experimental rat. Ramiprilat administration also reduced the myocardial infarct size in animal model [8, 26, 27].

The principle finding of the present is that there was increase in the levels of TBARS and decrease in the levels of SOD, catalase, and GSH. This indicates the development of oxidative stress. This is because the reperfusion of postischemic tissue is accompanied by the generation of large amount of oxygen free radicals formed by various mechanisms which can overwhelm the endogenous cellular defenses and induce tissue damage [28]. This was seen in ischemic control group when ischemia has been developed and then reperfused.

TBARS, one of the markers of oxygen free radical induced injury, has been used as a measure of lipid peroxidation when polyunsaturated fatty acids in myocardial cells are attacked by oxygen free radicals [29]. In the present study, TBARS level in ischemic control group was increased because of the oxidative stress. Ramipril, Candesartan, and combination of both the drugs lowered TBARS level. This decreased TBARS level in drug treated groups indicates that the given drugs inhibited the process of ROS induced lipid peroxidation and thus the oxidative stress mechanism.

Endogenous antioxidants like SOD, GSH, and catalase inhibit the generation of ROS and protect the myocardium from MIRI. SOD catalyses the dismutation of the highly reactive O_2_^•−^ anion into O_2_ and H_2_O_2_. This H_2_O_2_ on further reactions generate extremely reactive ^*∗*^OH radical. To inhibit this, GSH and catalase convert H_2_O_2_ into H_2_O and O_2_. Glutathione peroxides catalyse the peroxidation of H_2_O_2_ in the presence of reduced glutathione (GSH) to form H_2_O and oxidized glutathione (GSSG) [30].

The level of these endogenous antioxidants decreases in ischemic myocardium. In this present study, the SOD and GSH levels were significantly decreased in ischemic control group. Ramipril and Candesartan individually did not show much protective effect by reverting the decreased levels to normal, but the combination significantly reverted the levels of GSH and SOD to the normal. By observing this, increased endogenous antioxidant levels in combination treated group, monotherapy of the drugs exhibited less significant protective role against oxidative stress when compared to the combination treatment. In contrast to the above results, no significant effect was observed in case of catalase levels in the entire three drug treated groups. Many of the earlier studies results assessing the benefits of SOD alone or in combination with catalase in preventing reperfusion phenomena are conflicting [31].

Ramipril, a non-SH containing ACE inhibitor, inhibits free radical induced damage mainly by the stimulation of prostacyclin synthesis and or release which possess vasodilating membrane stabilizing properties and also decreases sodium accumulation, potassium loss, and intracellular calcium overload [32].

Although it is well established that AT_1_ receptor antagonists can protect against oxidative stress caused by Ang II, the findings of some studies indicate that some ARBs may also protect against intracellular oxidative stress induced by mechanisms other than Ang II-induced stimulation of AT_1_ receptors [33].

So, Ramipril being non-SH group containing ACE inhibitor alone and Candesartan alone did not produce significant reduction in oxidative stress. But both drugs in combination produced significant effect and it might be because of their additive inhibitory mechanism over the RAAS mechanism on inhibiting the activity of Ang II.

NO levels will get depleted during the ischemic conditions. Exogenous administered NO and endogenous NO may both play protective roles during ischemia and reperfusion injury. Protective actions of NO in ischemia and reperfusion are due to its potential as an antioxidant and anti-inflammatory agent, along with its beneficial effects on cell signaling and inhibition of nuclear proteins [34]. Administration of nitric oxide, NO donors, or drugs that enhance NO release (statins, calcium antagonists, ACE inhibitors, and dexamethasone) prior to ischemia protects the myocardium against MIRI [35].

The tissue nitrate levels were decreased significantly in ischemic control group when compared to that of control group due to ischemic injury. Ramipril did not improve the NO levels in the treated groups. Candesartan and combination increased the NO levels significantly when compared with ischemic control group. The protective activity of combination was higher when compared to Ramipril. Here, Ramipril failed to increase the tissue NO level. The increase in NO level in Candesartan and combination might be because of enhanced bradykinin levels and decreased attack of endogenous NO by the free radicals in the treated groups.

The role of ACE inhibitor and angiotensin receptor blocker drugs combination in MRI was further studied by the histopathology studies. Ramipril has a protective effect on isolated cardiac myocytes exposed to hypoxia/reoxygenation and that this effect is most likely related to a local action of bradykinin on the cardiac myocyte via the activation of the kinin B_2_ receptor [36]. AT_1_ receptor antagonist Candesartan reduces infarct size by angiotensin II type 2 receptor (AT_2_) activation, bradykinin, and prostaglandins [37].

In this study, it was found that there was extensive degeneration of myofibrils, edema, focal haemorrhage, and leukocyte infiltration which are indicative of necrosis in ischemic control group. The morphology of Ramipril, Candesartan, and combination groups on myocardial tissue was well preserved with branched appearance, continuity with adjacent myofibrils, and remained similar to that of control group. All three groups showed similar protective activity.

The extent of protective mechanism shown by Ramipril, Candesartan, and combination drugs on myocardial tissue was further studied by the TEM analysis which is an advanced method indicating the cellular damage at the mitochondrial level (ultrastructural changes). Characteristic changes like significant disruption of myofilament and Z-band architecture, loss of cell membrane integrity, interstitial edema, the appearance of vacuoles within the cell, and also changes in the mitochondrial architecture were seen in the rat heart subjected to MIRI. But myocardial ultrastructure was found to be well preserved and less evidence of myocyte injury was observed in Ramipril, Candesartan, and combination of both drugs treated groups with occasional disruption of myofilament and mild interstitial edema and less accumulation of electron dense material in mitochondria. The results of antioxidant studies, light microscopy study, and TEM analysis studies were correlated well and the antioxidant reports were well supported by the light microscopy and TEM analysis reports indicating that the combination has superior protective role compared to the monotherapy of individual drugs.

The limitation of present research is the effect of Ramipril and Candesartan on hemodynamic parameters and echo cardiography studies were not investigated. Further studies have to be conducted to study these effects to conform further mechanistic changes offered by these drugs.

## 5. Conclusion

In light of these findings, our study supports the hypothesis that Ramipril and Candesartan have protective role in myocardial ischemic reperfusion injury and justified their use in combination which has significant protective role in the treatment of ischemic heart diseases. Both drugs Ramipril and Candesartan when given alone as a monotherapy exhibited less significant protective activity, but they exhibited significant protective activity when were given in combination. Therefore, the protection against myocardial ischemic reperfusion injury in the combination treated rats is attributed to enhanced endogenous antioxidant activity and NO induced protective mechanism because of increased levels of NO which is due to the additive inhibitory effect on RAAS mechanism. Further studies may help to know the protective mechanisms of NO at the molecular level in salvaging the myocardial tissue against myocardial ischemic reperfusion injury.

## Figures and Tables

**Table 1:** Level of TBARS, GSH, SOD, and catalase in myocardial tissue.

S. number	Treatment	TBARS (nmol/gm wet wt)	GSH (*µ*g/gm wet wt)	SOD (IU/dL)	Catalase (IU/dL)
1	Group I	57.3 ± 10.3	222.1 ± 5.3	18.7 ± 6.3	21.73 ± 0.59
2	Group II	117.1 ± 36.8^##^	55.7 ± 5.9^##^	6.46 ± 0.13^#^	5.4 ± 0.55^##^
3	Group III	58.6 ± 1.1^*∗∗∗*^	61.04 ± 3.3^*∗∗*^	9.7 ± 4.9	6.04 ± 0.24
4	Group IV	52.2 ± 2.8^*∗∗∗*^	80.4 ± 10.2^*∗∗*^	13.4 ± 9.5	7.7 ± 1.03
5	Group V	43.6 ± 2.7^*∗∗∗*^	200.2 ± 8.1^*∗∗∗*^	26.4 ± 8.6^*∗∗∗*^	19.8 ± 0.78^*∗∗∗*^

All values were expressed as mean ± SEM, One-Way Analysis of Variance, followed by Dunnett's ^##^ *p* < 0.001 and ^#^ *p* < 0.05 versus Group I and ^*∗∗*^ *p* < 0.01, and  ^*∗∗∗*^ *p* < 0.001 versus Group II.

Group I: rats treated with saline (10 mL/kg p.o).

Group II: rats treated with saline (10 mL/kg p.o) and subjected to *in vitro* global ischemia

Group III: rats treated with Ramipril (2 mg/kg p.o) and subjected to *in vitro* global ischemia.

Group IV: rats treated with Candesartan (1 mg/kg p.o) and subjected to *in vitro* global ischemia.

Group V: rats treated with combination of both drugs and subjected to *in vitro* global ischemia.

**Table 2:** Nitrate level in myocardial tissue.

S. number	Group	Tissue nitrate level (*µ*g/dL)
1	Group I	52 ± 15.3
2	Group II	44 ± 2.3^#^
3	Group III	48 ± 3.7
4	Group IV	50 ± 3.6^*∗*^
5	Group V	55 ± 7.4^*∗∗∗*^

All values were expressed as mean ± SEM, One-Way Analysis of Variance, followed by Dunnett's ^#^ *p* < 0.01 versus Group I and ^*∗*^ *p* < 0.05,  and  ^*∗∗∗*^ *p* < 0.001 when compared with Group II.

Group I: rats treated with saline (10 mL/kg p.o).

Group II: rats treated with saline (10 mL/kg p.o) and subjected to *in vitro* global ischemia

Group III: rats treated with Ramipril (2 mg/kg p.o) and subjected to *in vitro* global ischemia.

Group IV: rats treated with Candesartan (1 mg/kg p.o) and subjected to *in vitro* global ischemia.

Group V: rats treated with combination of both drugs and subjected to *in vitro* global ischemia.
